# A systematic review of the effect of university positive psychology courses on student psychological wellbeing

**DOI:** 10.3389/fpsyg.2022.1023140

**Published:** 2022-11-15

**Authors:** Catherine Hobbs, Jessica Armitage, Bruce Hood, Sarah Jelbert

**Affiliations:** ^1^School of Psychological Science, University of Bristol, Bristol, United Kingdom; ^2^Wolfson Centre for Young People’s Mental Health, Cardiff University, Cardiff, United Kingdom

**Keywords:** positive psychology interventions, university, college, higher education, psychoeducation

## Abstract

Educational courses that teach positive psychology interventions as part of university degree programs are becoming increasingly popular, and could potentially form part of university-wide strategies to respond to the student mental health crisis. To determine whether such courses are effective in promoting student wellbeing, we conducted a systematic review of studies across the globe investigating the effects of positive psychology courses taught within university degree programs on quantitative measures of psychological wellbeing. We searched Embase, PsychInfo, PubMed, and Web of Science electronic databases from 1998 to 2021, identifying 27 relevant studies. Most studies (85%) reported positive effects on measures of psychological wellbeing, including increased life satisfaction and happiness. However, risk of bias, assessed using the ROBINS-I tool, was moderate or serious for all studies. We tentatively suggest that university positive psychology courses could be a promising avenue for promoting student wellbeing. However, further research implementing rigorous research practices is necessary to validate reported benefits, and confirm whether such courses should form part of an evidence-based response to student wellbeing.

**Systematic review registration:**

[https://www.crd.york.ac.uk/prospero/display_record.php?RecordID=224202], identifier [CRD42020224202].

## Introduction

Increasingly, concerns have been raised over the psychological wellbeing of university students. Psychological wellbeing encompasses both feelings of happiness (hedonic wellbeing), and a sense of meaning, purpose, or satisfaction with life (eudemonic wellbeing) (Deci and Ryan, 2008). A survey of over 10,000 students in the United Kingdom reported that just 11% report high levels of happiness and 6% report high life satisfaction (Neaves and Hewitt, 2021).

University students also commonly experience mental health issues, including suicidal ideation (Mortier et al., 2018). In an international survey, 31% of first-year students screened positive for a mental health disorder (Auerbach et al., 2018). This problem is expected to worsen with increasing numbers of young people entering university (Bolton, 2020). Although some researchers have proposed that wellbeing follows a ‘U’ shaped curve (Blanchflower and Oswald, 2008), reaching its lowest point during midlife, others’ have disputed this (Galambos et al., 2020).

Whilst poor psychological wellbeing in students may be partially due to the peak age of onset of mental health disorders coinciding with the average age of undergraduates (Kessler et al., 2005), wellbeing concerns are heightened among university students compared to peers (Lewis et al., 2021). Student psychological wellbeing declines after starting university, and does not return to pre-university levels (Bewick et al., 2010). Poor psychological wellbeing impairs academic performance (Bruffaerts et al., 2018) and increases the likelihood of dropping out (Hjorth et al., 2016). In contrast, positive psychological wellbeing increases confidence in completing degree programs (Lipson and Eisenberg, 2018).

At present, university services cannot adequately address students’ psychological wellbeing. Services are increasingly overburdened and under-resourced, resulting in long wait times (Randstad, 2020). One possible approach that may help to relieve these issues is to introduce academic courses focused on psychological wellbeing. Whereas traditional wellbeing services are targeted toward students with existing mental health difficulties, these courses take a community-wide approach, promoting psychological wellbeing at a university-level. Embedding these courses into degree programs is thought to be beneficial in promoting engagement and retention, as well as reducing stigma by having all students participate to meet course requirements.

University wellbeing courses take a variety of approaches, including mindfulness (Hassed et al., 2009), mental health literacy (Kurki et al., 2021), and psychoeducational life skills (Limarutti et al., 2021). In this review, we focus on wellbeing courses delivered within a positive psychology framework. Within such courses, students are taught evidence-based positive psychology interventions designed to enhance wellbeing by promoting happiness, life satisfaction, resilience, and social support. Examples include expressing gratitude (Wood et al., 2010), utilizing strengths (Quinlan et al., 2012), and performing acts of kindness (Curry et al., 2018).

Several meta-analyses have indicated that positive psychology interventions are effective in increasing psychological wellbeing and decreasing depression and anxiety, with benefits maintained at 3–6 months (Bolier et al., 2013; White et al., 2019; Carr et al., 2020). Within educational settings, the effectiveness of courses teaching positive psychology interventions has been predominantly investigated in schools (Seligman et al., 2009). Systematic reviews have concluded that positive psychology courses delivered as part of primary and secondary school curriculum benefit students’ psychological wellbeing, mental health, and academic performance (Waters, 2011; Tejada-Gallardo et al., 2020).

The strong evidence base underlying positive psychology interventions suggests that they may be beneficial in promoting student psychological wellbeing when delivered as part of university curriculum. Such courses are increasingly common (see Barrington-Leigh, 2022 for a list of courses offered across universities), are highly popular, and receive widespread media attention (Shimer, 2018). However, there is a lack of systematic understanding of the nature of these courses and their effects on psychological wellbeing. With courses reaching growing numbers of students, it is increasingly important that we understand their impact.

As there was a conspicuous gap in the literature, we therefore conducted a systematic review of wellbeing courses teaching positive psychology interventions that were embedded in university degree programs. Systematic reviews are an essential component of research governance and good professional practice. We aimed to identify the characteristics of courses offered to students, and whether the courses have a positive effect on psychological wellbeing and mental health.

## Methods

### Systematic review protocol

This review was pre-registered on PROSPERO prior to study searches being conducted^1^.

### Inclusion criteria

Inclusion criteria for studies was formulated using the PICOS framework as follows:

• *Participants* were students enrolled in taught degree programs at higher education institutions.
• *Interventions* were courses embedded within university taught degree programs that used positive psychology techniques with the aim of improving student psychological wellbeing.
• *Comparators* were other higher education courses without positive psychology interventions. For studies without control conditions, we included studies comparing within-subject effects from pre- to post-course.
• *Outcomes* were quantitative measures of psychological wellbeing. As a secondary outcome we extracted data for quantitative measures of mental health difficulties. We included loneliness within this due to strong links with mental health (Mann et al., 2022).
• *Study Designs* were restricted to quantitative studies, although studies could use a variety of designs including randomized clinical trials, quasi-experimental and observational designs.

We restricted studies to those available in English and published from 1998 onward, to coincide with the beginning of the positive psychology movement as in similar reviews (Bolier et al., 2013).

### Exclusion criteria

We excluded (1) narrative reviews, systematic reviews, and meta-analyses, (2) conference proceedings/presentations as sufficient data was typically not available to establish eligibility, (3) studies that were not available in English, (4) courses that only implemented mindfulness-based techniques as there is already well-established evidence of the benefits of such courses in university settings (Dawson et al., 2020). During the screening stage, we also chose to exclude courses that only taught Acceptance and Commitment Therapy (ACT) techniques. Although ACT incorporates Positive Psychology principles it originated from a clinical psychology framework (Howell and Passmore, 2019). Additionally, the effectiveness of ACT courses in university settings has previously been reviewed (Howell and Passmore, 2019).

### Search strategy

We searched four electronic databases for studies published between January 1998 and November 2021: PsychInfo, PubMed, Embase, and Web of Science. We used four main search terms combined using the Boolean operator ‘And’:

(1) Wellbeing OR wellbeing OR ‘positive psychology’ OR happiness OR happy OR ‘PERMA’
(2) University OR college OR ‘higher education’ OR undergraduate
(3) Course OR programme OR program
(4) Effect OR impact OR eval* OR effic*

### Study screening and selection, data extraction and study quality assessment

The references of studies identified in the electronic search were uploaded to the systematic review software Covidence, and duplicates were removed. The following steps were then completed independently by two reviewers, and disagreements at each stage were resolved by a third reviewer. Studies were first screened based on titles and abstracts, and then re-assessed based on the full text. The reference list of identified studies were manually searched for additional eligible studies, which underwent the same screening procedure. A standardized, pre-piloted form was used to extract data.

Reviewers also independently conducted a quality assessment using the risk of bias in non-randomized studies of interventions (ROBINS-I) tool (Sterne et al., 2016). Potential bias relating to confounding, participant selection, classification of interventions, deviations from intended interventions, missing data, measurement of outcomes, and selection of reported results was assessed. An overall risk of bias was determined based on the highest risk identified in each subtype of risk.

## Results

### Search

**FIGURE 1:**
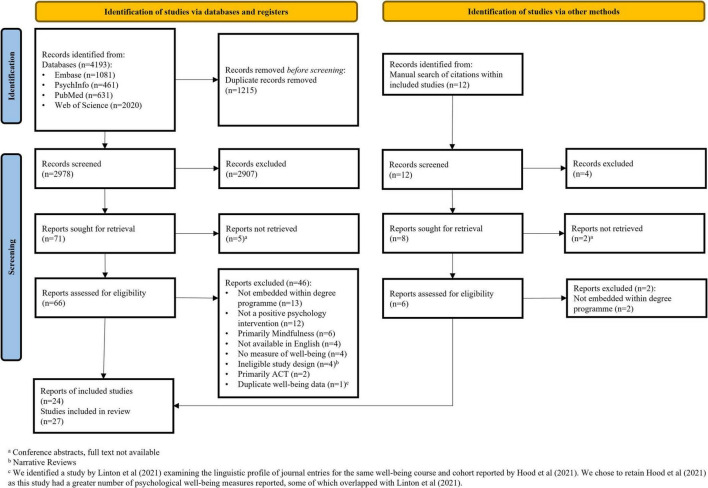
PRISMA flow diagram illustrating the study search process.

Figure 1 shows a PRISMA flow diagram detailing the full search process. A simplified table illustrating the search results is presented in Supplementary Table 1. From 2978 unique references identified in electronic databases and 12 references from manual searches, 27 studies were included. Inter-rater reliability was κ = 0.58 for title and abstract screening, and κ = 0.72 for the full text screening.

### Study characteristics

**FIGURE 2:**
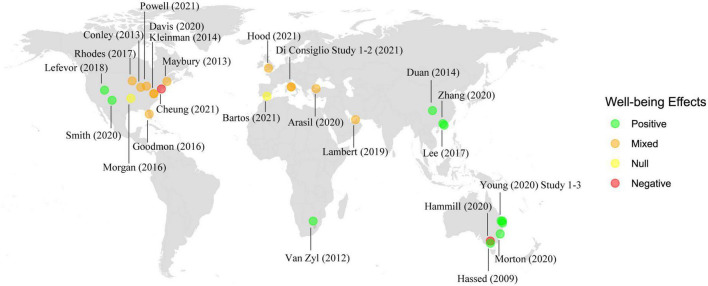
Summary of study effects on psychological well-being by geographic location.

Study characteristics are summarized in Table 1. Studies were published between 2009 and 2021 and were conducted in 10 countries (Figure 2). The most common countries were the USA (*k* = 11), Australia (*k* = 6), China (*k* = 2), and Italy (*k* = 2).

**TABLE 1:** Study characteristics and reported effects of interventions on wellbeing.

Study	Institution, country	Timepoints	Intervention condition					Control condition			Well-being	
			N Pre	N Post	Course length	Program	PPIs	Type	N Pre	N Post	Measure	Effect
Arasil et al., 2020	Üsküdar University, Turkey	Pre, Post	308	308	14 weeks	Mandatory open unit	Empathy, emotional skills, communication skills, relationships, resilience	None	–	–	Life evaluation question^a^	Increase
											WEMWBS	None
											OHQ	None
											SWLS	None
											PWI	None
Bartos et al., 2021	Royal Conservatory of Music Victoria Eugenia, Spain	Post	82	40	27 weeks	Music	Gratitude, kindness, empathy, strengths, mindfulness, physical activity (yoga), emotional skills, communication skills, flow	Other elective course^b^	115	53	VAS psychological health	None
Cheung et al., 2021	Albert Einstein College of Medicine, USA	Each session	157	157	16 weeks	Medicine	Gratitude, kindness, savoring, mindfulness, emotional skills, positive reappraisal	None	–	–	Frequency of positive emotions	Decrease
Conley et al., 2013	Loyola University Chicago, USA	Pre, Post	29	29	8 months	Open unit	Strengths, emotional skills, communication skills, relationships, stress management	Alternative course (‘Global citizens and citizenships’)	22	22	Latent variable ‘positive wellbeing’	None
											Perceived improvements – Psychosocial adjustment	Increase
Davis, 2020	James Madison University, USA	Pre, Post	30	30	15 weeks	Psychology	Gratitude, forgiveness, savoring, strengths, mindfulness, physical activity, emotional skills, engaging with the natural environment, resilience, meaning in life, flow, positive reappraisal, relationships	Other psychology course	20	20	Henrique’s 10-Item wellbeing scale	Increase
											PANAS positive	Increase
											PERMA profiler	None
											SLS	None
											OHQ	None
Di Consiglio et al., 2021, Study 1	Sapienza University of Rome, Italy	Pre, Post	12	12	12 weeks^c^	Open Unit	Gratitude, kindness, empathy, strengths, emotional skills, communication skills	Offline version of unit without exercises and self-monitoring tools	12	12	R-PWB	Increase in self-acceptance subscale only
Di Consiglio et al., 2021, Study 2	Sapienza University of Rome, Italy	Pre, Post	154	59	12 weeks^c^	Open Unit	Gratitude, kindness, empathy, strengths, emotional skills, communication skills	None	–	–	R-PWB	Increase in self-acceptance subscale only
Duan et al., 2014	Southwest University, Chongqing, China	Pre, Post, 18-weeks	211	211	6 weeks^d^	Psychology writing skills training	Strengths	Psychology writing skills training without positive psychology interventions^e^	74	74	SWLS	Increase
Goodmon et al., 2016	Florida Southern College, USA	Pre, Post	18	18	16 weeks	Psychology	Gratitude, kindness, forgiveness, savoring, strengths, mindfulness	Social psychology course	20	20	AHI	Increase
											GHQ	None
											SWLS	Increase
											AHQ	Increase
Hammill et al., 2020	Victoria University, Australia	Post	37	37	4 weeks	Business	Gratitude, strengths, mindfulness	Comparator course^f^	21	21	Likert scale – Happy at university	No change in intervention group vs. increase in control croup
Hassed et al., 2009	Monash University, Australia	Pre, Post	239	148	6 weeks	Medicine	Mindfulness, physical activity, stress management, meaning in life, relationships	None	–	–	WHOQOL psychological	Increase
Hood et al., 2021	University of Bristol, UK	Pre, Post, 6-weeks	135	119	12 weeks	Open unit	Gratitude, kindness, savoring, strengths, mindfulness, physical activity	Wait-list	137	118	SWEMWBS	Increase
											ONS Life Satisfaction	None
											ONS life worthwhile	No change in intervention group vs. small decrease in control group
											ONS Happiness	None
Kleinman, 2014 ^g^	James Madison University, USA	Pre, Post, 4-months	25	17	14 weeks	Psychology	Kindness, forgiveness, strengths, mindfulness, physical activity, meaning in life, emotional skills, relationships, resilience, stress management	Other psychology course	26	14	PWBNF	None
											SWLS	None
											PANAS Positive	None
											OHQ	None
											WBI	Increase
											R-PWB	None
Lambert et al., 2019	Canadian University Dubai, UAE	Pre, Post, 3-months	159	159	14 weeks	Open unit	Gratitude, forgiveness, savoring, mindfulness	‘Not enrolled in the course’^f^	108	108	SPANE	None
											SWLS	None
											Flourishing scale	None
											QEWB	Increase
											MHC-SF	None
Lee, 2017	School of Continuing Education of Hong Kong Baptist University, Hong Kong	Pre, Post	44	44	12 weeks	Professional Diploma in Applied Psychology	Gratitude, kindness, empathy, savoring, strengths, mindfulness, relationships, meaning in life	Other psychology course	50	50	SHS	Increase
											SWLS	Increase
											AHS	Increase
Lefevor et al., 2018	Brigham Young University, USA	Pre, Post	146	133	One semester^h^	Open unit	Gratitude, savoring, strengths, mindfulness	None	–	–	R-PWB	Increase
											LOT-R	Increase
											AHQ	Increase^i^
											SHS	Increase
Maybury, 2013	University of Maine at Farmington, USA	Pre, Post	32	23	14 weeks	Open unit	Gratitude, strengths	None	–	–	SWLS	None
											THS	Increase
											SHS	Increase
Morgan, 2016	University of Arkansas, USA	Pre, Post	53	53	8 weeks	Public health	Gratitude, kindness, forgiveness, strengths, mindfulness, communication skills	Personal health and safety course	22	22	QEWB	None
Morton et al., 2020	Avondale College of Higher Education, Australia	Pre, Post	67	67	10 weeks	Open unit	Gratitude, kindness, forgiveness, mindfulness, physical, emotional skills, communication skills, engaging with the natural environment, relationships	None	–	–	SWLS	Increase
Powell et al., 2021	University of Michigan, USA	Pre, Post	42	38	15 weeks	Pharmacy	Kindness, mindfulness	None	–	–	Brief inventory of thriving	Increase only ‘Life having a sense of purpose’ item
Rhodes, 2017	Walden University, USA	Pre, Post	25	25	9 weeks	Foundational courses at a non-traditional career college	Gratitude, kindness, strengths	None	–	–	AHI	Small increase
											SWLS	None
Smith et al., 2020	University of New Mexico, USA	Pre, Post	127	112	16 weeks	Psychology	Gratitude, kindness, strengths	Other psychology courses^j^	325	176	PERMA profiler	Increase
Van Zyl and Rothmann, 2012	North-West University, South Africa	Pre, Post, 4-months	20	20	8 months	Industrial/Organizational psychology	Gratitude, forgiveness, savoring, strengths, mindfulness, positive visualization, relationships, communication skills	None	–	–	SWLS	Increase
											PANAS affect balance	Increase
Young et al., 2020, Study 1	University of Queensland, Australia	Weekly during course	67	38	6 weeks	Psychology	Gratitude, kindness, strengths, mindfulness, positive visualization, emotional skills	None	–	–	MHC-SF	Increase
Young et al., 2020, Study 2	University of Queensland, Australia	Pre, Post	155	129	6 weeks	Psychology	Gratitude, kindness, strengths, mindfulness	None	–	–	MHC-SF	Increase
											PANAS positive	Increase
Young et al., 2020, Study 3	University of Queensland, Australia	Pre, Post	105	55	6 weeks	Psychology	Gratitude, kindness, strengths, mindfulness	Other psychology course	83	58	MHC-SF	Decrease in control, no change in intervention
Zhang et al., 2020	South China University of Technology, China	Pre, Post	113	95	8 weeks	Medicine	Gratitude, forgiveness, strengths, meaning in life, emotional skills, relationships, resilience, stress management	None	–	–	THS	Increase
											SWLS	Increase
											SHS	Increase

AHI, Authentic Happiness Inventory; AHQ, Approaches to Happiness Questionnaire; AHS, Adult Hope Scale; GHQ, General Happiness Questionnaire; LOT-R, Life Orientation Test-Revised; MHC-SF, Mental Health Continuum Short-Form; OHQ, Oxford Happiness Questionnaire; PANAS, Positive and Negative Affect Schedule; PERMA Profiler, Positive Emotion, Engagement, Relationships, Meaning, and Accomplishment Profiler; PPIs, Positive Psychology Interventions; PWBNF, Psychological Well-Being Narrative Form; PWI, Personal Wellbeing Index; QEWB, Questionnaire for Eudemonic Well-Being; R-PWB, Ryff’s Scales of Psychological Well-Being; SHS, Subjective Happiness Questionnaire; SWEMWBS, Short Warwick Edinburgh Mental Wellbeing Scale; SWLS, Satisfaction with Life Scale; SPANE, Scale of Positive and Negative Experience; THS, Trait Hope Scale; WBI, The Well-Being Interview; WEMWBS, Warwick Edinburgh Mental Wellbeing Scale, WHOQOL Psychological, World Health Organization Quality of Life.

^a^Single item question: ‘How do you rate yourself when you think about your whole life in general?’ Responses ranged from (1) very unhappy to (5) very happy.

^b^Other elective courses included History of Spanish Music, Ergonomics, Ethnomusicology, Ensemble Foundations of Direction, German, English.

^c^Students completed the course online in their own time, the maximum permitted time for completion was 12 weeks.

^d^Total course was 18 weeks, but positive psychology component was only included for 6 weeks.

^e^Participants in the control condition were asked to write about 10 things they had done that week instead of the positive psychology intervention.

^f^No further details provided.

^g^Thesis version of the study included in this review as it included a greater number of well-being measures. A shortened version was also published in an academic journal (Kleinman et al., 2014).

^h^Length of the semester not reported.

^i^A significant increase was observed in the ‘meaning’ subscale, change in the ‘pleasure’ and ‘engagement’ were trend level effects (*p* = 0.053).

^j^Alternative psychology courses included were Cognitive Psychology, Statistics, Neuropsychology, Psychology of Perception, Research Methods.

Thirteen studies used a within-subjects design, measuring change in wellbeing pre- and post-course. Twelve studies used a mixed-design, comparing change in wellbeing pre- and post-course in intervention and control groups. Two studies used a between-subject design, comparing psychological wellbeing post-course only in intervention and control groups.

Most studies collected psychological wellbeing outcomes at two timepoints, pre- and post-course (*k* = 18). Five studies collected additional follow-up timepoints varying from 6 weeks to 4 months. Two studies collected psychological wellbeing measures at each session, and two studies collected post-course timepoints only.

### Participants

A total of 2,176 participants were analyzed as part of intervention groups across studies (mean = 81, *SD* = 71, min = 12, max = 308). However, demographics were reported inconsistently. Where participant characteristics were reported, participants tended to be young (reported mean range of 18.8–25.9) and predominantly female (reported range of 49–92%). Of studies reporting ethnicity, most students were caucasian except for two studies that reported an equal or greater number of hispanic students (Rhodes, 2017; Smith et al., 2020).

### Intervention groups – positive psychology courses

#### Course features

Courses were embedded into a variety of programs, including open units available to all students (*k* = 9), Psychology (*k* = 10), Medicine (*k* = 3), Business (*k* = 1), Music (*k* = 1), Pharmacy (*k* = 1), Public Health (*k* = 1), and Foundational courses (*k* = 1). Courses ranged from 4 weeks to 8 months, with a median length of 12 weeks.

#### Positive psychology interventions

The most common positive psychology interventions taught within courses focused on character strengths (*k* = 21) and gratitude (*k* = 21). Activities related to character strengths included identifying strengths and using strengths in new ways. Gratitude activities included writing messages of gratitude to others and keeping a gratitude journal.

Eighteen studies taught activities relating to mindfulness. This involved meditation practices and mindful listening. Sixteen studies covered acts of kindness, where, most commonly, students were asked to do something nice for someone else without expecting anything in return. Eight studies included activities or readings relating to forgiveness. Most commonly, students were asked to write a letter forgiving a previous transgressor. Eight studies covered savoring, such as focusing on sensations during an enjoyable activity (e.g., eating chocolate). Five studies included components related to empathy, such as empathetic listening. Finally, five studies included physical health components, such as completing 30 min of exercise per day.

Beyond the positive psychology interventions extracted in accordance with our protocol, we identified several other topics including emotional skills training (e.g., emotional intelligence, awareness and regulation; *k* = 12), communication skills (e.g., conflict management; *k* = 8), promoting social relationships (e.g., reflecting on how one’s relationships impact wellbeing; *k* = 9), spirituality and meaning in life (*k* = 5), positive visualization (e.g., drawing a picture of a positive future; *k* = 3), stress management and resilience (e.g., coping strategies; *k* = 6), engaging with the natural environment (e.g., immersing oneself in a brightly lit natural environment; *k* = 2), and positive reappraisal (e.g., positively reframing stressful events, *k* = 2).

### Control groups

Half of studies (*k* = 14, 52%) used a control group, none of which randomized group assignment. Ten studies compared positive psychology courses to alternative university courses; six were alternative Psychology courses, one a Personal Health and Safety course, one a Global Citizens and Citizenships course, one any elective course, and one study did not provide details of the comparison course. Two studies used the same type of course in both intervention and control groups but removed the positive psychology interventions for the control group. One study used a wait-list control group of students enrolled to take the course in the following academic semester. One study did not provide details of the nature of the comparison group. Wellbeing was analyzed in a total of 768 participants in control groups across studies (mean = 55, *SD* = 49, min = 12, max = 176).

### Outcome measures

#### Psychological well-being

In total, 33 different measures of psychological wellbeing were used (Table 1). On average, studies used two measures of psychological wellbeing (Min = 1, Max = 6). The most common measure was the Satisfaction with Life Scale (*k* = 12). Most studies used validated measures of psychological wellbeing. However, three studies used single-item Likert or Visual Analog Scales only (Hammill et al., 2020; Bartos et al., 2021; Cheung et al., 2021). One study used ten individual measures of wellbeing to create a latent variable of ‘positive wellbeing’ (Conley et al., 2013).

#### Mental health difficulties

Of studies included in the review, thirteen studies also assessed the impact of positive psychology courses on mental health. Of these, an average of two measures were included per study (Min = 1, Max = 4). This included measures of depression, anxiety, stress, loneliness, and burnout (Table 2). However, there was little overlap in measures across studies.

**TABLE 2:** Summarized effects of positive psychology courses on measures relating to mental health.

Area of mental health	Study	Measure	Effect
Anxiety	Bartos et al., 2021	Change in anxiety (yes/no)	None
	Di Consiglio et al., 2021, Study 2	Anxiety sensitivity index	None
		Social interaction anxiety scale	None
		Social phobia scale	None
	Hassed et al., 2009	Symptom checklist revised	None
	Hood et al., 2021	ONS anxiety	None
	Morton et al., 2020	DASS	Decrease
	Zhang et al., 2020	PROMIS	Decrease
Burnout	Cheung et al., 2021	Modified Maslach burnout inventory	Increase
Depression/Negative affect	Cheung et al., 2021	Frequency of negative emotions	None
	Conley et al., 2013	Negative distress latent variable	None
	Davis, 2020	PANAS negative	None
		PERMA negative emotion	None
	Goodmon et al., 2016	Center for epidemiologic studies depression questionnaire	Decrease
	Hassed et al., 2009	Symptom checklist revised	Decrease
	Kleinman, 2014	PANAS negative	Decrease ^a^
	Morton et al., 2020	DASS	Decrease
	Smith et al., 2020	PERMA negative emotion	Decrease
	Zhang et al., 2020	PROMIS	Decrease
Stress	Bartos et al., 2021	Change in stress (yes/no)	None
	Cheung et al., 2021	Perceived stress scale	None
	Conley et al., 2013	Perceived improvements in stress scale	Decrease ^b^
	Goodmon et al., 2016	Perceived stress scale	Decrease
	Morton et al., 2020	DASS	Decrease
Loneliness	Davis, 2020	PERMA loneliness	None
	Hood et al., 2021	ONS loneliness	Decrease
		UCLA 3-item loneliness scale	None
	Smith et al., 2020	PERMA loneliness	Decrease
Worry	Di Consiglio et al., 2021, Study 2	Penn state worry questionnaire	Decrease
Overall psychological distress	Bartos et al., 2021	Mental emotional issues (yes/no)	None
	Hassed et al., 2009	Symptom checklist revised	Decrease
	Lefevor et al., 2018	Outcome questionnaire-45	Decrease

DASS, Depression, Anxiety, and Stress Scale; PANAS, Positive and Negative Affect Scale; PERMA, Positive Emotion, Engagement, Relationships, Meaning, and Accomplishment Profiler; ONS, Office of National Statistics.

^a^Weak evidence, group × time interaction effect of *p* = 0.051.

^b^Represented by an increase in scores.

### Effect on psychological wellbeing

Results are summarized in Table 1 and Figure 2. Eleven (41%) studies reported positive effects across all measures of psychological wellbeing, 12 (45%) studies reported positive findings on at least one measure of psychological wellbeing but also reported null effects, two (7%) studies reported null effects on all measures, and two (7%) studies reported negative effects.

#### Positive effects

Eleven studies (41%) reported consistently beneficial effects of positive psychology courses across all measures of psychological wellbeing employed. Three of these studies reported a relatively greater increase in psychological wellbeing in the intervention versus control group (Duan et al., 2014; Lee, 2017; Smith et al., 2020). One study reported a decline in emotional wellbeing in the control group versus stable levels in the intervention group, suggesting the course may have had a protective effect (Young et al., 2020).

Seven studies reported an increase in psychological wellbeing from pre- to post-course but did not include a control group comparison. Of these, two were delivered to medical students (Hassed et al., 2009; Zhang et al., 2020), three to psychology students (Van Zyl and Rothmann, 2012; Young et al., 2020), and two as open units (Lefevor et al., 2018; Morton et al., 2020).

#### Mixed effects

Twelve studies (44%) reported at least one positive effect across measures of psychological wellbeing. However, the extent of supportive evidence varied. Providing stronger support that the course had a beneficial effect, Goodmon et al. (2016) reported increases in life satisfaction and two measures of happiness relative to a control group, but did not find an effect for a third measure of happiness. Similarly, Maybury (2013) reported increases in hope and happiness, but not life satisfaction.

Providing weaker evidence of beneficial effects, Davis (2020) found evidence of increased positive mood and wellbeing measured using Henrique’s 10-item wellbeing scale, but no evidence of positive effects on three other measures of wellbeing. Similarly, Hood et al. (2021) reported increased wellbeing using the Shortened Warwick Edinburgh Mental Wellbeing Scale (SWEMWBS) in students completing an open-unit wellbeing course versus no change in a wait-list control group. Additionally, whereas the wait-list control group showed a decline in feelings that life was worthwhile, no change was found in the intervention group. However, no evidence of beneficial effects were reported for measures of happiness or life satisfaction. Kleinman (2014) reported increased wellbeing when measured using a structured clinical assessment (the wellbeing interview), but did not find evidence in five self-report measures of wellbeing. Arasil et al. (2020) used five measures of wellbeing, reporting only positive effects in a single-item Likert scale question developed for the study, but not for the four validated measures of wellbeing. Conley et al. (2013) found evidence of greater perceived wellbeing but only when measured post-course. No evidence of a beneficial effect was found for a latent measure of ‘positive wellbeing’ collected pre- and post-course. Lambert et al. (2019) reported increased eudemonic wellbeing but no effects for four other measures. Di Consiglio et al. (2021) reported only beneficial effects on a single subscale of Ryff’s Psychological Wellbeing scale across two studies. Rhodes (2017) reported only weak evidence of a change in happiness and no change in life satisfaction. Lastly, in a pilot study of pharmacy students, Powell et al. (2021) found beneficial effects on only a single item of the Brief Inventory of Thriving (‘Life having a sense of purpose’).

#### Null and negative effects

Two studies (7%) reported no evidence of a difference in wellbeing between intervention and control groups (Morgan, 2016; Bartos et al., 2021). Two studies (7%) reported negative effects. Business students that completed the positive psychology course showed lower levels of happiness compared to the control group (Hammill et al., 2020). Medicine students reported a decline in the frequency of positive emotions throughout the course (Cheung et al., 2021).

#### Long term effects

Five studies examined psychological wellbeing at follow-up timepoints. Duan et al. (2014) found that the participants in the intervention group continued to show greater life satisfaction versus controls. Hood et al. (2021) reported that psychological wellbeing effects were maintained 6-weeks post-course, but benefits were no longer found for perceptions that life was worthwhile. Van Zyl and Rothmann (2012) and Lambert et al. (2019) found that wellbeing increased from pre-course to three and four month follow-ups respectively. However, for Lambert et al. (2019) these analyses were conducted in the intervention group only, limiting conclusions. Finally, Kleinman (2014) reported no differences between intervention and control groups at 4-month follow-up. However, an abbreviated battery of measures was completed, none of which showed an effect post-course.

### Effect on mental health

Most studies investigating mental health reported beneficial effects on at least one measure (*k* = 10; 77%) (Hassed et al., 2009; Conley et al., 2013; Kleinman, 2014; Goodmon et al., 2016; Lefevor et al., 2018; Morton et al., 2020; Smith et al., 2020; Zhang et al., 2020; Di Consiglio et al., 2021, Study 2; Hood et al., 2021). However, when examining individual areas of mental health, findings were more mixed (Table 2). Positive effects were observed in 2/6 (33%) studies examining anxiety (Morton et al., 2020; Zhang et al., 2020), 6/9 (66%) studies examining depression/negative affect (Hassed et al., 2009; Kleinman, 2014; Goodmon et al., 2016; Morton et al., 2020; Smith et al., 2020; Zhang et al., 2020), and 3/5 (60%) studies examining stress (Conley et al., 2013; Goodmon et al., 2016; Morton et al., 2020). Two of three studies reported beneficial effects on loneliness (Smith et al., 2020; Hood et al., 2021). However, in one of these studies, there was disagreement between the different measures of loneliness; although a decline in loneliness was observed in the intervention group using the single-item ONS measures, no change was found using the UCLA loneliness scale (Hood et al., 2021). One study reported negative effects on mental health, with medical students reporting increased burnout throughout the course (Cheung et al., 2021).

### Characteristics of courses with a positive effect on psychological wellbeing

Benefits to psychological wellbeing were observed across studies with varying characteristics, including participants’ degree programs, lengths of courses, and positive psychology interventions taught. We therefore did not identify specific characteristics of courses that may be linked to positive effects.

### Risk of bias

**FIGURE 3:**
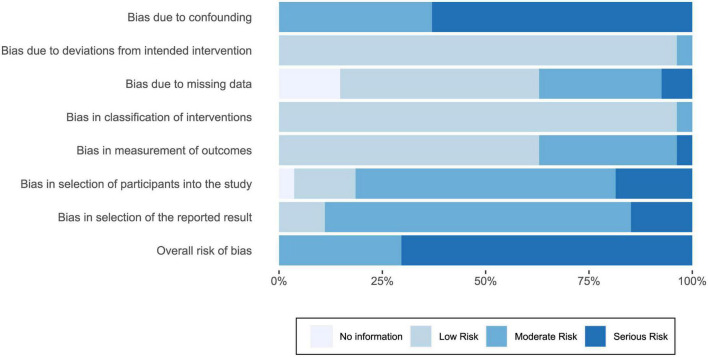
Summary of risk of bias judgments, illustrating the proportion of studies that were judged to be at low, moderate, or serious risk of bias (or whether sufficient information was not available for a judgment) for individual domains of bias and overall risk.

Figure 3 summarizes judgments of risk for each domain of bias. Individual judgments per study are available in Supplementary Table 2. Across studies, overall risk of bias was moderate (*k* = 8; 30%) or serious (*k* = 19; 70%). Most studies were at high risk of bias as they did not adjust for potential confounding effects in statistical analyses, such as imbalanced characteristics between groups. Studies were also at moderate or serious risk of bias in the selection of reported results, with only one study pre-registering statistical analyses (Hood et al., 2021). However, all studies included in the review followed the intended intervention. Additionally, bias in the classification of interventions and measurement of outcomes was mainly low.

## Discussion

There is increasing concern over university students’ psychological wellbeing (Auerbach et al., 2018; Neaves and Hewitt, 2021). Positive psychology courses embedded into university degree programs may help address this problem. Such courses have received positive media attention (e.g., Shimer, 2018), but to date evidence of their effectiveness has not been systematically established. We therefore conducted a systematic review of positive psychology courses embedded into university degree programs.

We found evidence that the majority of studies evaluating such courses reported beneficial effects on student psychological wellbeing, including increased levels of happiness, life satisfaction, and quality of life. Of the 27 studies included in this review, 23 (85%) studies reported at least one positive effect, of which 11 reported consistently positive findings across all psychological wellbeing measures employed. However, risk of bias was high across studies. Findings are therefore not conclusive but suggest that positive psychology courses delivered within degree programs may be one tool to promote psychological wellbeing in university students.

Thirteen studies also examined the impact of courses on measures relating to mental health difficulties. Although psychological wellbeing courses are not intended as a treatment for mental health disorders, there was some evidence across this subset of studies that courses were beneficial in reducing mental health difficulties including depression, negative affect, and stress. Implementing courses into university curriculum may be valuable in supporting students with mental health difficulties until services designed to treat these problems are available.

However, it must be acknowledged that there was also evidence of null and negative effects of courses on wellbeing. Forty-four percent of studies reported mixed findings across different measures of wellbeing used. Two studies reported null effects, and of most concern, two studies reported negative effects on student wellbeing. One study found that business students reported lower levels of happiness compared to controls (Hammill et al., 2020). However, this study used only a single-item Likert scale to measure happiness and did not provide details of the nature of the comparator group. Additionally, one study found that medicine students reported reduced positive emotions and increased burnout (Cheung et al., 2021). As this study lacked a control group this effect may be attributed to the wider stressors of the degree program. However, other courses in medicine students reported positive effects suggesting that positive psychology courses can be effective in similar degree programs (Hassed et al., 2009; Zhang et al., 2020).

It is possible that differences in findings between studies was partly attributable to substantial variation across courses and study designs. There was no positive psychology intervention that was implemented across all courses, although character strengths and gratitude were most common. Courses varied in length, ranging from as brief as 4 weeks to as long as 8 months. Additionally, there was also inconsistency in measures used to evaluate psychological wellbeing. Although most studies used validated self-report measures, some studies focused on single-item measures developed for the study, making comparisons problematic. Due to these variations it is difficult to identify aspects of courses that may be most beneficial or detrimental to participants, or whether effects may be more sensitively detected using certain measures.

Furthermore, study quality was generally poor with most studies at high risk of bias. In particular, studies were at moderate or high risk of bias for selective reporting of results. Only one study identified in this review pre-registered hypotheses or statistical analyses (Hood et al., 2021), and there was also inconsistency between reported outcomes in methods and results, increasing the likelihood of false-positive findings (Simmons et al., 2011). Additionally, most studies did not control for potential confounding. The beneficial effects of courses highlighted in this review may therefore be at least partly attributable to poor research practices in this field.

### Future research and recommendations

The findings from our review raise several questions regarding the use of positive psychology courses within student populations. Firstly, the magnitude of effects is unclear. It is possible that whilst these courses have a statistically significant effect on psychological wellbeing, these changes are small and not perceived as meaningful by students (Hobbs et al., 2021). Future studies should ensure outcome data are fully reported, or where possible data is published open access to allow meta-analysis of effects. This would also allow the use of meta-regression to identify which aspects of courses are most beneficial to student wellbeing.

Secondly, it is currently unclear as to what extent positive findings may be attributable to poor quality research in this area. Future studies within this field should implement rigorous research practices to validate the reported beneficial effects of positive psychology courses. In light of our findings, we recommend researchers pay greater consideration to potential confounding effects, as well as pre-registering hypotheses and statistical analyses to ensure validity of findings.

Additionally, we identified that there is currently large variation in the type of positive psychology courses on offer across universities, making comparisons problematic. It is likely that the quality of courses and the type of positive psychology interventions on offer moderate effects on student wellbeing. Future reviews in this field should consider including a measure of the quality of courses to evaluate this possibility.

We also identified substantial variation in study designs. One source of variation that would be relatively easy to address in future research is identifying a consistent self-report measure of wellbeing to use across studies. This would enable clearer comparisons of course effectiveness. One potential candidate may be the Satisfaction with Life Scale (Diener et al., 1985) as this was the most widely used measure across studies, has good psychometric properties (Pavot et al., 1991), and is relatively brief.

Finally, it is somewhat unclear how long beneficial effects of courses on psychological wellbeing are sustained for. Whilst four studies found some evidence that effects were sustained approximately 4 months post-course, most studies did not conduct follow-ups. It would be helpful for studies to implement long-term follow-ups with participants, using a short self-report measure, to help estimate how long effects of courses may be maintained for. In the future, this may aid the development of brief ‘top-up’ interventions to further sustain potential beneficial effects of courses on wellbeing.

### Strengths and limitations

Despite the growing popularity of university positive psychology courses (Shimer, 2018), this is the first systematic review on the effects of these courses on psychological wellbeing. Taking a rigorous approach, from approximately 3,000 unique records we identified 27 studies in this field, documenting effects for each measure of wellbeing employed.

However, this review was limited in its focus on quantitative measures of wellbeing. Whilst this allowed us to compare quantified effects on wellbeing, we lack a detailed understanding of students’ experiences of participating in such courses, which may be better obtained using a qualitative approach.

Additionally, despite focusing on quantitative measures we were unable to conduct a meta-analysis due to studies not reporting sufficient data. We are therefore not able to comment on the magnitude of effects or conduct meta-regressions that may allow us to more precisely identify beneficial course aspects.

We did not extract data on the quality of courses on offer or student engagement. It is likely that these factors play a role in the effect of courses on student wellbeing. Future reviews in this field should consider including this information to determine where courses may be most beneficial for students.

Finally, we combined findings using different measures of wellbeing. Our findings are therefore based on the assumption that effects from different measures are comparable despite measuring distinctive facets of wellbeing. However, as this is the first systematic review in this field, it was not possible to anticipate which wellbeing measure would be most appropriate to extract. Future reviews in this field focusing on specific measures would be helpful in confirming our findings.

## Conclusion

From systematic review of current literature, we found that most studies report at least one positive effect of positive psychology courses embedded within university degree programs on student psychological wellbeing. However, a small number of studies report null or negative effects. A lack of consistency across courses on offer makes it difficult to determine which aspects of courses are most beneficial for students. Furthermore, study quality was relatively poor, increasing the potential for false-positive findings and potential confounding. Adoption of rigorous research practices, including pre-registration and open-access publication of data, is required to confirm the positive effects of positive psychology courses embedded into university degree programs on student psychological wellbeing.

## Data availability statement

The original contributions presented in this study are included in the article/Supplementary material, further inquiries can be directed to the corresponding author.

## Author contributions

CH, BH, and SJ conceptualized the study. CH wrote the review protocol, which was reviewed and edited by JA, BH, and SJ. CH conducted the database search. CH and JA screened the studies, extracted the data, and assessed risk of bias. SJ acted as a third reviewer and resolving disagreements. CH wrote the original draft of this manuscript, which was reviewed and edited by JA, BH, and SJ. All authors contributed to the article and approved the submitted version.

## Footnotes

1
https://www.crd.york.ac.uk/prospero/display_record.php?RecordID=224202
