# Supplementary material for: A systematic review of the effect of university positive psychology courses on student psychological wellbeing
Source: Front Psychol. 2022 Nov 15;13:1023140. doi: 10.3389/fpsyg.2022.1023140 (PMC9705334; doi:10.3389/fpsyg.2022.1023140)
Supplement: Supplementary file 1 [file Table_1.docx]

**Supplementary information for Hobbs, C., Armitage, J., Hood, B., Jelber, S. A systematic review of the effect of university positive psychology on student well-being.**

Supplementary Table 1

*Study Search Results*

| **Stage** | **Records** |
| --- | --- |
| Records Identified (with duplicates removed) | 4205 |
| Records Screened | 2990 ^a^ |
| Reports Assessed for eligibility | 72 ^b^ |
| Studies included in review | 27 |
| Risk of bias judgements (overall risk) | Serious: 19  Moderate: 8 |

^a^ 98% agreement between reviewers

^b^ 87% agreement between reviewers

Supplementary Table 2

*Risk of bias judgements per study*

|  | **Bias Category** | | | | | | | |
| --- | --- | --- | --- | --- | --- | --- | --- | --- |
| **Study** | **Confounding** | **Selection of participants** | **Classification of interventions** | **Deviations from intended intervention** | **Missing data** | **Measurement of outcomes** | **Selection of the reported result** | **Overall risk** |
| Arasil (2020) | Serious | Serious | Low | Low | No info. | Low | Moderate | Serious |
| Bartos (2021) | Serious | Serious | Low | Moderate | Moderate | Serious | Moderate | Serious |
| Cheung (2021) | Serious | Low | Low | Low | No info. | Moderate | Moderate | Serious |
| Conley (2013) | Serious | Low | Low | Low | Low | Low | Moderate | Serious |
| Davis (2021) | Serious | Moderate | Low | Low | No info. | Moderate | Moderate | Serious |
| Di Consiglio (2021) Study 1 | Serious | Moderate | Low | Low | Moderate | Moderate | Moderate | Serious |
| Di Consiglio (2021) Study 2 | Serious | Moderate | Low | Low | Moderate | Moderate | Moderate | Serious |
| Duan (2014) | Moderate | Moderate | Low | Low | No info. | Low | Moderate | Moderate |
| Goodmon (2016) | Moderate | Low | Low | Low | Low | Low | Moderate | Moderate |
| Hammill (2020) | Serious | No info. | Low | Low | Serious | Moderate | Moderate | Serious |
| Hassed (2009) | Serious | Moderate | Low | Low | Moderate | Moderate | Moderate | Serious |
| Hood (2021) | Serious | Low | Low | Low | Low | Moderate | Low | Serious |
| Kleinman (2014) | Moderate | Moderate | Low | Low | Serious | Moderate | Serious | Serious |
| Lambert (2019) | Moderate | Moderate | Moderate | Low | Moderate | Low | Moderate | Moderate |
| Lee (2018) | Moderate | Moderate | Low | Low | Low | Low | Moderate | Moderate |
| Lefevor (2018) | Serious | Moderate | Low | Low | Low | Low | Moderate | Serious |
| Maybury (2013) | Serious | Serious | Low | Low | Moderate | Low | Moderate | Serious |
| Morgan (2016) | Moderate | Moderate | Low | Low | Low | Low | Moderate | Moderate |
| Morton (2020) | Serious | Moderate | Low | Low | Low | Low | Moderate | Serious |
| Powell (2021) | Serious | Serious | Low | Low | Low | Moderate | Serious | Serious |
| Rhodes (2017) | Serious | Moderate | Low | Low | Low | Low | Moderate | Serious |
| Smith (2020) | Moderate | Moderate | Low | Low | Moderate | Low | Moderate | Moderate |
| Van Zyl (2012) | Serious | Moderate | Low | Low | Low | Low | Serious | Serious |
| Young (2021) Study 1 | Moderate | Moderate | Low | Low | Low | Low | Low | Moderate |
| Young (2021) Study 2 | Moderate | Moderate | Low | Low | Low | Low | Low | Moderate |
| Young (2021) Study 3 | Moderate | Moderate | Low | Low | Low | Low | Serious | Serious |
| Zhang (2020) | Serious | Serious | Low | Low | Moderate | Low | Moderate | Serious |
